# Flexible Capacitive Electrodes for Minimizing Motion Artifacts in Ambulatory Electrocardiograms

**DOI:** 10.3390/s140814732

**Published:** 2014-08-12

**Authors:** Jeong Su Lee, Jeong Heo, Won Kyu Lee, Yong Gyu Lim, Youn Ho Kim, Kwang Suk Park

**Affiliations:** 1 Interdisciplinary Program for Bioengineering, Graduate School, Seoul National University, Seoul 110-799, Korea; E-Mails: dynamicjs@snu.ac.kr (J.S.L.); hjeong20@bmsil.snu.ac.kr (J.H.); wongyu86@bmsil.snu.ac.kr (W.K.L.); 2 Department of Oriental Biomedical Engineering, Sangji University, Wonju 220-702, Korea; E-Mail: yglim@sangji.ac.kr; 3 Samsung Advanced Institute of Technology, 130, Samsung-ro, Yeongtong-gu, Suwon 443-803, Korea; E-Mail: yh92.kim@samsung.com; 4 Department of Biomedical Engineering, College of Medicine, Seoul National University, Seoul 110-799, Korea

**Keywords:** capacitive electrode, flexible electrode, ambulatory electrocardiogram

## Abstract

This study proposes the use of flexible capacitive electrodes for reducing motion artifacts in a wearable electrocardiogram (ECG) device. The capacitive electrodes have conductive foam on their surface, a shield, an optimal input bias resistor, and guarding feedback. The electrodes are integrated in a chest belt, and the acquired signals are transmitted wirelessly for ambulatory heart rate monitoring. We experimentally validated the electrode performance with subjects standing and walking on a treadmill at speeds of up to 7 km/h. The results confirmed the highly accurate heart rate detection capacity of the developed system and its feasibility for daily-life ECG monitoring.

## 1. Introduction

The periodic measurement of physiological signals is one of the best methods for detecting the occurrence and observing the progress of disease. Many studies have proposed daily monitoring systems for measuring body signals such as electrocardiograms (ECGs), ballistocardiograms (BCGs), photoplethysmograms (PPGs), body temperature, blood pressure, and daily activity [1–6]. ECGs are some of the most important, most studied, and most widely used signals for screening cardiovascular diseases (CVDs) and evaluating heart or cardiovascular functions [7–9].

The conventional ECG measurement method can only be implemented in a hospital environment. Special equipment and several wired direct-skin-contact Ag/AgCl electrodes are required, and it is not suitable for long-term daily-life ECG monitoring because of the skin irritation it causes, occurrence of motion artifacts, and disturbance of daily life activities caused by the wired electrodes. To overcome these drawbacks, several studies have developed alternative monitoring systems. Dry electrodes and non-direct contact methods have been proposed to reduce skin irritation [10–12]. Sensors integrated into home and office furniture and appliances such as chairs, toilet seats, baths, computer mice, and beds have been suggested to facilitate unconstrained long-term monitoring [2,4,13,14]. Wearable medical devices, which have the advantage of ubiquitously acquiring physiological signals, have also been proposed for daily physiological monitoring. The development of wearable medical devices has been boosted by the prevalence of smartphones and related applications [7,15,16].

With regard to reducing skin irritation and realizing unconstrained monitoring, the use of capacitive coupling electrodes is considered a promising solution. Such electrodes can acquire ECG signals through clothing without the use of a conductive gel or direct skin contact. Several types of capacitive coupling electrodes have been developed. Lim *et al.* attached an electrode to the back of a chair and a mattress [2,13]. Aleksandrowicz *et al.* also applied such electrodes to a chair [17]. Nemati *et al.* integrated electrodes into a cotton T-shirt [18]. As noted above, many studies have developed various methods for ambulatory ECG measurement. However, practical ambulatory acquisition of valuable long-term data for predicting the occurrence or observing the progress of disease requires the elimination or minimization of motion artifacts. Thus far, no study has developed a robust strategy to eliminate motion artifacts or to establish their effects on a capacitive electrode system in a real-life environment. Lee *et al.* introduced a belt-type flexible capacitive electrode but did not validate its performance in daily life [19]. They only used it to measure the ECG signals when the subjects swung their body backward and forward, and observed that bigger electrodes increased the gain and signal-to-noise ratio. However, increasing the electrode size also increased motion artifacts owing to the large unstable contact area.

Hence, in the present study, we reduced the size of the capacitive electrode and focused on achieving a robust design that minimizes motion artifacts. Specifically, we designed an ambulatory ECG monitoring system with coupled capacitive electrodes that were embedded in a chest belt with a wireless system. Wearing comfort should be considered when designing the wearable medical device. We therefore designed the electrodes to be flexible, particularly adopting strategies for reducing the effect of motion artifacts. Conductive foam was used to reduce the air gap between the flexible electrode and the body surface and to minimize the unstable contact caused by motion artifacts. The foam was also used as a shield or buffer from external motion artifacts. Optimal input bias resistance was used to minimize the motion artifact. Materials that ensured flexibility and satisfied the other requirements were adopted. To validate the feasibility of the developed system for ambulatory ECG monitoring, we applied it to subjects standing and walking on a treadmill at speeds of up to 7 km/h.

## 2. Flexible Electrode

### 2.1. Structure of the Flexible Electrodes

**Figure 1.**
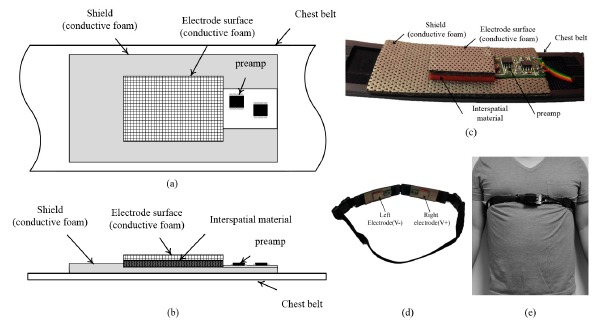
Configuration of the flexible electrode: (**a**) Top view; (**b**) side view; (**c**) photograph of actual electrode; (**d**) overall design; (**e**) person with the system around the waist.

When developing wearable medical devices for long-term monitoring, user comfort should be considered. To minimize the duration of data acquisition disruptions caused by motion artifacts, the device should be worn sufficiently tightly. However, we observed that rigid electrodes left marks on the skin of subjects even when the electrode was worn on clothing, and there were complaints about discomfort. We therefore designed a flexible electrode, as shown in Figure 1. The electrode comprised the electrode surface, a preamp circuit, a shield, and an interspatial material between the electrode surface and the shield. The shield is positioned between the interspatial material and the chest belt. A photograph of the electrode is shown in Figure 1c.

#### 2.1.1. Electrode Surface

**Figure 2.**
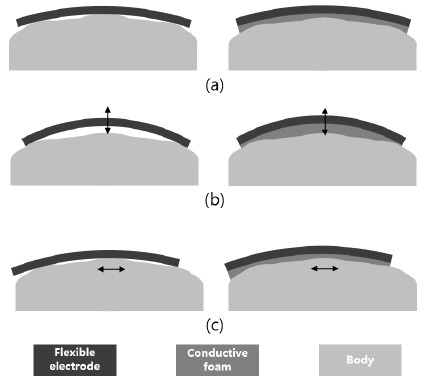
(**a**) A conventional flexible electrode bends along the body curvature as shown in the left diagram, whereas a flexible electrode with conductive foam also covers the air gaps as shown in the right diagram; (**b**) Lifting of electrode caused by motion artifacts; (**c**) Sliding of electrode surface in the direction of motion artifacts (clothing is omitted in this figure).

Normally, an electrode surface is made from copper or gold-coated copper. In this study, conductive foam was attached to the electrode surface. The foam was produced from polyolefin covered by polyurethane. To make the foam conductive, the entire material surface was coated with Ni/Cu [20]. The electrode was 1.1 ± 0.2 mm thick, and its surface resistance was less than 0.08 Ω/sq. A conventional flexible electrode bends and adapts to the curvature of the chest, as shown in the left diagram in Figure 2a. However, there are air gaps between the electrode and the body owing to the uneven surface of the chest caused by the ribs. The conductive foam adopted in the present study eliminates the air gaps, as shown in the right diagram in Figure 2a. Furthermore, the occurrence of motion artifacts, which causes unstable contact between the body surface and the electrode, critically weakens the effectiveness of the wearable device for physiological signal measurement. To minimize the effect of unstable contact on the signal measurement, the foam was applied as shown in the right diagrams in Figure 2b,c.

#### 2.1.2. Shield Material

The conductive foam was also used to shield the flexible electrode. In this case, the foam functioned as an automobile bumper to minimize external motion artifacts introduced through the belt. The size of the shield was 80 × 35 mm, and the thickness was 2.0 mm. The shield was designed to cover the entire electrode surface and the preamp circuit when worn by the subjects. The exterior of the shield was attached to the belt, as shown in Figure 1.

#### 2.1.3. Interspatial Material

In a rigid capacitive electrode, the shielding plate is made from rigid aluminum or copper and is attached at a fixed height from the electrode face. The space between the electrode surface and the shield is filled by air (dielectric constant = 1.00059) or rigid materials such as glass resin or a printed circuit board (PCB) [21,22]. In the presently developed flexible electrode, the interspatial material was used to maintain a fixed distance between the electrode surface and the shield. The material was required to be flexible but noncompressible in the thickness direction. Compressibility in the thickness direction, which would make *d* in Equation (1) variable, would cause variation of the stray capacitance between the electrode surface and the shield when motion artifacts occur, resulting in fluctuation of the total gain of the electrode in Equation (3). Secondly, the interspatial material was required to have a low dielectric constant (*ε_r_* in Equation (1)) to minimize the stray capacitance:

(1) $$C_{\text{stray}}={ɛ}_{r}{ɛ}_{0}\frac{A}{d}$$

Among five candidate materials that satisfied the above requirements, we selected urethane rubber as the interspatial material based on the results of a preliminary study.

### 2.2. Front-End Circuit

**Figure 3.**
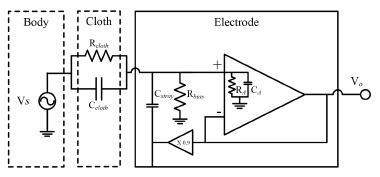
Overall equivalent circuit of the electrode.

The overall equivalent electrical circuit of the electrode is shown in Figure 3. The gain of the circuit is given by:

(2) G(s)s=VoVs=ZB//ZAZC+ZB//ZA,if|ZA|≫|ZB|,thenZAcan be ignored

where the symbol // indicates the parallel combination of two impedances, *Z_A_* is the input impedance of the operational amplifier (R*_A_*//C*_A_*), *Z_B_* is the impedance of the parallel combination of *C_stray_* and *R_bias_*, and *Z_C_* is the impedance of the cloth (R*_cloth_*//C*_cloth_*). Because *Z_A_* is much greater than *Z_B_, Z_A_* can be ignored. The gain can thus be expressed as:

(3) $$G_{s}(s)=\frac{R_{B}+sC_{\text{cloth}}R_{\text{bias}}R_{\text{cloth}}}{(R_{\text{bias}}+R_{\text{cloth}})+s(0.9C_{\text{stray}}C_{\text{cloth}})R_{\text{bias}}R_{\text{cloth}}}$$

#### 2.2.1. High-Input Impedance Amplifier

An operational amplifier with a high-input impedance is required to acquire ECG signals through the insulation of clothing. OPA124 (TI, Dallas, TX, USA), which has an input impedance of 10^13^ Ω ‖ 1 pF, was selected for use as the preamplifier.

#### 2.2.2. Bias Resistor

**Figure 4.**
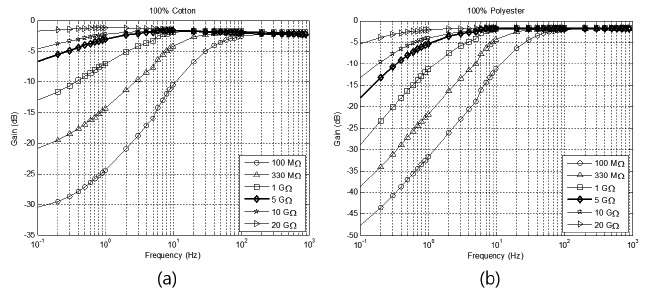
Frequency responses for different resistances: (**a**) Using 100% cotton as insulator; (**b**) using 100% polyester as insulator.

The bias resistor was used to provide a path of biased current to the preamp. A very high resistance of the order of gigaohms was used in previous studies owing to the high impedance of clothing [2,17,19]. To determine the most appropriate value of the resistance, we performed simulations using various values between 100 MΩ and 20 GΩ. Five fabrics were used for the simulation, namely, 100% cotton of 0.30-mm thickness, polyester of 0.45-mm thickness, crepe of 0.4-mm thickness, silk of 0.18-mm thickness, wool of 0.25-mm thickness, and denim of 0.2-mm thickness. The room temperature and humidity were controlled to 20 °C and 50%, respectively. Two (cotton and polyester) among the six fabrics were worn by each subject during the walking experiments. As shown in Figure 4, the value of the resistance determined the cut-off frequency of the high-pass filter produced by the combination of the clothing capacitance and the resistor. Based on the results, a 5 GΩ resistor was adopted. A resistance of below 1 GΩ would not be suitable for ECG measurement because it would attenuate the ECG signals, which have a main bandwidth of 0.5–35 Hz. A resistance higher than 5 GΩ would also not be suitable for reducing motion artifacts because the main bandwidth of the artifact is below 5 Hz [23].

#### 2.2.3. Guarding Feedback

An increase in the stray capacitance (*C_stray_*) reduces the total gain of the front-end circuit as indicated by Equation (3). To produce a thin electrode, which would increase the stray capacitance, the distance between the electrode surface and the shield was made as small as possible. Furthermore, to minimize the effect of the increased stray capacitance, the guarding feedback was connected to the shielding fabric instead of the ground, as done by Lee [19].

#### 2.2.4. Driven Right-Leg (DRL) Electrode

To minimize the common-mode interference of the subjects during ECG measurement, the driven right-leg (DRL) electrode method was adopted in the system [24]. Moreover, to achieve full nondirect-contact measurement, the driven electrode was also not allowed to make direct contact with the skin. The conductive foam was used as a stable conduit of the signal between the driven electrode and the body even in the presence of motion artifacts, as described in Section 2.1.1. The common-mode voltage was generated by an operational amplifier (INA118). The signal was high-pass-filtered at the cut off frequency of 0.5 Hz to reduce the time constant after saturation of the amplifier due to motion artifacts. The filtered signal was amplified by an inverting amplifier with a gain of 100, and the amplified signal was delivered to the subject through the driven electrode. The electrode had a size of 300 × 20 mm and was attached to the chest belt such that it would be positioned on the left side of the back of the subject.

### 2.3. Overall System

**Figure 5.**
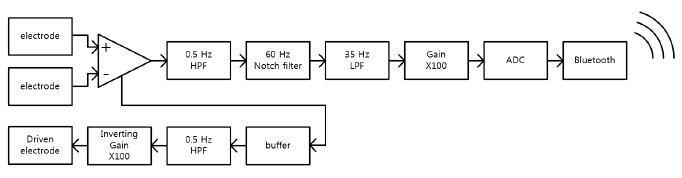
Schematic of the overall system.

A schematic of the overall system is shown in Figure 5. The signals acquired through the two flexible electrodes are amplified by an instrumentation amplifier (INA118, TI), and the output signal is filtered and further amplified as illustrated in the diagram. The signal is then digitalized at a sampling rate of 256 Hz by a microcontroller (ATmega128, Atmel, San José, CA, USA). Finally, the signal is transmitted through a Bluetooth module (Parani-ESD200, SENA, San José, CA, USA) to a personal computer.

## 3. Experiments

### 3.1. Participants

Four healthy male subjects aged between 28 and 32 years participated in the experiment, all of whom gave prior informed written consent. None of them suffered from a cardiovascular disease or was undergoing any medical treatment.

### 3.2. Experimental Procedure

We acquired the ECG signals from the subjects while they were wearing the belt on their chest and standing or walking on a treadmill. The belt was sufficiently tightened without complaint of discomfort. The participations were asked to stand at rest on the ground for 3 min. They were thereafter requested to walk on the treadmill at speeds of 4, 5, 6, and 7 km/h for 3 min each. Simultaneous ECG measurements were performed using the proposed system and a Biopac MP150 (Biopac Inc., Goleta, CA, USA) with a conventional Ag/AgCl electrode. The latter measurement was used as a reference.

### 3.3. Signal Processing

The performance of the proposed system was verified by comparing its measured heart rate with that of the reference system. To extract the heart rate, we used a self-designed automatic peak detection algorithm for QRS complex detection and a MATLAB peak modification program. For precise peak extraction, the acquired signal was filtered by a band-pass filter at a cut-off frequency of 15–35 Hz.

## 4. Results and Discussion

### 4.1. Bias Resistor

Figure 4 shows the obtained frequency responses for two fabrics and different resistances. As can be seen, the value of the resistance determined the cut-off frequency of the high-pass filter. Based on these results, we selected a resistance of 5 GΩ. Although higher resistances increased the unit gain of the system, they were not suitable for attenuating motion artifacts. Moreover, resistances lower than 5 GΩ attenuated the ECG signal. These phenomena are described in detail in Section 2.2.2.

### 4.2. Experiment

**Figure 6.**
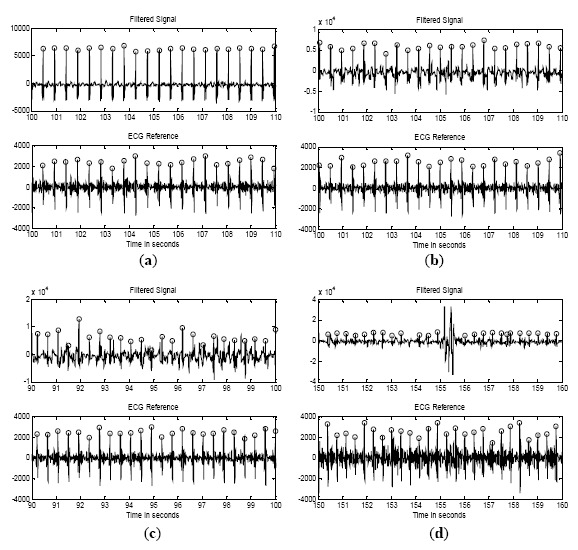
Signals acquired at different walking speeds: (**a**) 4 km/h; (**b**) 5 km/h; (**c**) 6 km/h; (**d**) 7 km/h (top: proposed system, bottom: reference system).

Figure 6 shows the signals acquired from subject 1 while walking at the specified speeds. At each walking speed, his arm naturally swung as he walked. As shown in the figure, only QRS complex was dominantly observed and T and P waves were attenuated because the acquired signal was filtered as described in Section 3.3. At a speed of 4 km/h, vivid peaks were observed as shown in Figure 6a. False positive peaks or true negative peaks were seldom observed. At a speed of 5 km/h, the base line noise in the filtered signal increased as shown in Figure 6b owing to the drift in the base line of the raw signal. More false positive peaks and true negative peaks occurred at a speed of 6 km/h, as can be observed at around 98 s in Figure 6c. Missing data owing to saturation of the amplifier caused by excessive movement of the arms or body were observed at a speed of 7 km/h, as shown in Figure 6d. To validate the system performance, we considered two statistical indices, namely, the sensitivity and the overall accuracy, which were determined as follows:

(4) $$\text{Sensitivity}(\%)=\frac{\text{TP}}{\text{TP}+\text{FN}}\times 100$$

(5) $$\text{Accuracy}(\%)=\frac{\text{TP}}{\text{TP}+\text{FP}+\text{FN}}\times 100$$

where *TP* represents true positive, which is a quantification of the correctly detected QRS complex; *FN* represents false negative, which is a quantification of the missed QRS complex; and *FP* represents false positive, which is a quantification of the noise spikes detected by our peak detection algorithm. The results for the four subjects are presented in Table 1. As indicated, there was no false-positive peak or false-negative peak when any of the subjects was at rest. However, the number of false detections increased with the walking speed. It was also observed that the wetting of clothing by sweat at a fast speed reduced the resistance of the cloth and enhanced the signal-to-noise ratio. The results for subject 3, who sweated more, confirm this. The data in Table 1 indicates that the accuracy at 7 km/h was higher than that at 6 km/h. On average, including the results for the highest walking speed of 7 km/h, the system had a high QRS complex detection accuracy of 91.32%.

## 5. Conclusions

In this study, we have proposed a wearable device for ambulatory ECG monitoring. When developing a wearable device, user comfort, usability, stability for long-term monitoring, and the reliability of acquired signals should be considered. To enhance user comfort, we designed a flexible capacitive electrode that could bend along the curvature of the body surface. The electrode surface was covered with conductive foam to further enhance user comfort; the foam also served to shield the electrode. The interspatial material was chosen to satisfy the flexibility requirements. The capacitive coupled method enables an ECG signal to be measured over cloth. Subjects can easily wear the system on their clothes and take it off without any skin preparation or taking off their clothes. Therefore, in terms of usability, the proposed system is more advantageous. To minimize motion artifacts caused in daily life monitoring, we proposed several strategies. To minimize instability at the electrode contact owing to motion artifacts, the electrode surface was covered with conductive foam; the foam also served as a shield to reduce external motion artifacts introduced through the belt. A bias resistor was further used to reduce the effect of motion artifacts. A 5 GΩ resistor was specifically chosen to eliminate the bandwidth of motion artifacts. However, the result shown in this study can vary with the clothing material and thickness, contact pressure, and external environment such as atmospheric humidity. Moreover, the appropriate bias resistor value can also vary with the clothing because the cut-off frequency of the high-pass filter was observed to be a function of the capacitance of the clothing and bias resistance.

The present study shows the feasibility of the wearable system for ambulatory ECG monitoring. To enhance the system performance, the qualities of the peak detection and noise cancelation algorithms should be enhanced. In a future study, the proposed system will be used for 24-h heart rate monitoring during daily life to evaluate the dynamics of its autonomic function by heart rate variability (HRV) analysis.

## Figures and Tables

**Table 1.** Statistical results for QRS complex detection.

**Subject**	**Status**	**TP (Beats)**	**FP (Beats)**	**FN (Beats)**	**Sensitivity (%)**	**Accuracy (%)**
Subject 1	Standing	361	0	0	100	100
	4 km/h	377	0	0	100	100
	5 km/h	469	2	3	99.36	98.95
	6 km/h	422	9	15	96.59	94.62
	7 km/h	464	30	54	89.58	84.67

Subject 2	Standing	280	0	0	100	100
	4 km/h	315	3	3	99.06	98.13
	5 km/h	325	5	4	98.78	97.31
	6 km/h	341	8	7	97.99	95.79
	7 km/h	400	12	15	96.39	93.68

Subject 3	Standing	479	0	0	100	100
	4 km/h	375	3	0	100	99.21
	5 km/h	406	8	5	98.78	96.90
	6 km/h	434	16	11	97.53	94.55
	7 km/h	350	10	6	98.31	95.62

Subject 4	Standing	239	0	0	100	100
	4 km/h	277	3	1	99.74	98.58
	5 km/h	288	5	14	95.36	93.81
	6 km/h	304	9	17	94.70	92.12
	7 km/h	347	15	18	95.07	91.32

Mean	Standing	339.75	0	0	**100**	**100**
	4 km/h	336	2.25	1	**99.7**	**98.98**
	5 km/h	372	5	6.5	**98.07**	**96.74**
	6 km/h	375.25	10.5	12.5	**96.70**	**94.27**
	7 km/h	390.25	16.75	23.25	**94.84**	**91.32**
